# Association and predictive value of systemic immune–inflammation index and body mass index in cervical ossification of the posterior longitudinal ligament

**DOI:** 10.3389/fmolb.2026.1823906

**Published:** 2026-05-08

**Authors:** Tingxiao Zhao, Shanggao Xie, Yanlei Li, Liangjian Xu, Kaifeng Ding, Yazeng Huang, Xijing He

**Affiliations:** 1 Department of Orthopedics, The Second Affiliated Hospital of Xi’an Jiaotong University, Xi’an Jiaotong University, Xi’an, Shaanxi, China; 2 Center for Plastic and Reconstructive Surgery, Department of Orthopedics, Zhejiang Provincial People’s Hospital (Affiliated People’s Hospital, Hangzhou Medical College), Hangzhou, Zhejiang, China; 3 Emergency and Critical Care Center, Department of Emergency, Zhejiang Provincial People’s Hospital (Affiliated People’s Hospital, Hangzhou Medical College), Hangzhou, Zhejiang, China; 4 Department of Orthopedics, Xi’an International Medical Center Hospital, Xi’an, Shaanxi, China

**Keywords:** body mass index, cervical ossification of the posterior longitudinal ligament, predictive value, risk factors, SII, systemic immune-inflammation index

## Abstract

**Introduction:**

Cervical ossification of the posterior longitudinal ligament (C-OPLL) is a common cause of cervical myelopathy. While mechanical and metabolic factors have been implicated in its pathogenesis, the role of systemic inflammation remains unclear. This research aimed to explore the link between inflammation-related biomarkers and C-OPLL and their predictive ability.

**Methods:**

A total of 442 patients (211 C-OPLL, 231 controls) were enrolled in this study. We collected demographic data, comorbidities, and preoperative blood parameters. The calculation of Systemic immune–inflammation index (SII) and other inflammatory indices was performed. Independent risk factors were identified through multivariate logistic regression. Subgroup and interaction analyses assessed the combined effect of body mass index (BMI) and SII on C-OPLL.

**Results:**

Patients with C-OPLL showed significantly higher BMI and SII levels than controls (all p < 0.05). Multivariate logistic regression analysis indicated that SII (odds ratio [OR]: 1.121; 95% confidence interval [CI]: 1.101–1.210; P < 0.01) and BMI (OR: 1.412; 95% CI: 1.251–1.594; P < 0.01) were independent predictors of C-OPLL. The AUC for SII on the ROC curve was 0.82. The SII demonstrated a sensitivity of 73.0% and a specificity of 80.5% at a cutoff of 464.2, derived from the current dataset using Youden index analysis. Subgroup analyses consistently showed a positive association between SII and C-OPLL, with no significant interactions detected. A significant synergistic effect was observed between obesity and high SII (P = 0.038). Obese patients with high SII had the highest prevalence of C-OPLL (63.1%) and the greatest risk (OR = 6.23, compared with normal-weight individuals with low SII). Both SII and BMI were independently associated with C-OPLL.

**Conclusion:**

These findings suggest that metabolic burden and systemic inflammatory activation may be involved in C-OPLL pathogenesis. Inflammation-based measures such as SII could therefore complement risk stratification, though further research is needed.

## 1 Introduction

Ossification of the posterior longitudinal ligament (OPLL) is a common spinal disorder characterized by ectopic ossification within the posterior longitudinal ligament, most commonly at the cervical spine region, and often resulting in compression of spinal cord and nerve roots (Okubo et al., 2025). The prevalence of cervical OPLL (C-OPLL) in the East Asian population is high (Sun et al., 2024). According to Fujimori et al., the prevalence was 6.3% in a Japanese population while Liang et al. reported a prevalence around 4.1% in a Chinese population from their CT-based analysis of 2000 individuals (Fujimori et al., 2016; Liang et al., 2019). C-OPLL is a prime cause of cervical myelopathy probably showing up as neck pain, constrained cervical motion, limb numbness, and both motor and sensory dysfunction (Mun et al., 2024). As ossification progresses gradually and often remains asymptomatic in the early stages, diagnosis is frequently delayed until significant spinal canal compromise develops. Moreover, patients with advanced OPLL are particularly vulnerable to catastrophic neurological deterioration following minor trauma, potentially resulting in quadriplegia, substantial impairment of quality of life, and increased mortality risk (Wang et al., 2025; Mori et al., 2025). Although surgical decompression is still the most effective treatment for symptomatic C-OPLL, the procedure is technically challenging and involves significant perioperative risks (Ramos et al., 2021). Therefore, identifying modifiable risk factors and clinically accessible biomarkers is essential for early diagnosis and the development of preventive strategies.

C- OPLL is considered a multifactorial disorder involving genetic predisposition, senescence, metabolic imbalance, obesity, chronic inflammation, and mechanical stress (Singh et al., 2024; Fukada et al., 2023). Advanced age and elevated body mass index (BMI) have been identified as independent risk factors for C-OPLL according to previous studies (Hisada et al., 2022; Katsumi et al., 2023). Endo et al. showed a significant association between OPLL and BMI regardless of symptoms, but this association was stronger for cervical involvement (Endo et al., 2022). Besides, biomechanical factors were discovered to contribute to the progression of the disease. *Ex vivo* studies revealed that ligament-derived cells from C-OPLL patients exhibit heightened sensitivity to mechanical stress stimulation, and repetitive tensile loading has been reported to upregulate osteogenic gene expression and increase alkaline phosphatase activity, thereby promoting ectopic ossification (Iwasawa et al., 2006; Sawada et al., 2008). These findings suggest that local mechanical stress, along with systemic metabolic factors, plays a role in aberrant osteogenic differentiation in the posterior longitudinal ligament.

Emerging evidence indicates that systemic inflammation is pivotal to the development and progression of several non-immunological diseases, including tumours, intervertebral disc degeneration, heterotopic ossification (Huang et al., 2021; Chen et al., 2017; Zhou et al., 2018; Eraslan Doganay and Cirik, 2022). A number of composite indices were developed to quantify systemic inflammatory status from circulating leukocyte subpopulations such as neutrophil-to-lymphocyte ratio (NLR), platelet-to-lymphocyte ratio (PLR), monocyte-to-lymphocyte ratio (MLR), systemic immune–inflammation index (SII) (Gao et al., 2019; Jiang et al., 2026; Rinawati et al., 2026). They have demonstrated prognostic and diagnostic utility across numerous diseases, including various cancers, type 2 diabetes, and acute ischemic stroke (Jiang et al., 2026; Rinawati et al., 2026; Yan et al., 2020). Notably, SII has been recognized as an independent risk factor for ossification of the ligamentum flavum (Zhao et al., 2022). Nevertheless, the relationship between systemic inflammatory damage markers and C-OPLL has yet to be systematically analyzed.

Given the potential mechanistic link between chronic inflammation and aberrant ossification, we hypothesized that systemic inflammatory indices may be associated with C-OPLL. Therefore, we aimed to systematically investigate the correlation between multiple inflammatory biomarkers and the risk of C-OPLL occurrence, assess their independent predictive value, and provide evidence for early clinical identification of high-risk individuals and research into disease mechanisms.

## 2 Methods

### 2.1 Study design and ethical approval

This retrospective study was approved by the Ethics Committee of Zhejiang Provincial People’s Hospital (Approval No: QT2025422). Given the retrospective nature of the study, the requirement for informed consent was waived.

### 2.2 Study population

**FIGURE 1:**
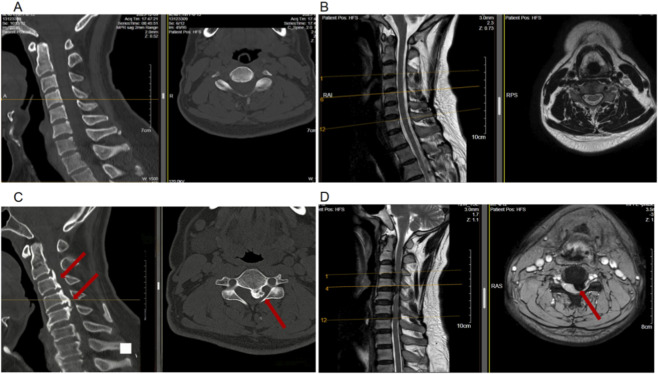
None-Ossification of the posterior longitudinal ligament in Cervical spine CT scan **(A)**, MRI scan **(B)**,and the spinal cord within the spinal canal is not compressed; Ossification of the posterior longitudinal ligament (red arrow) in Cervical spine CT scan **(C)**, MRI scan **(D)**.

**FIGURE 2:**
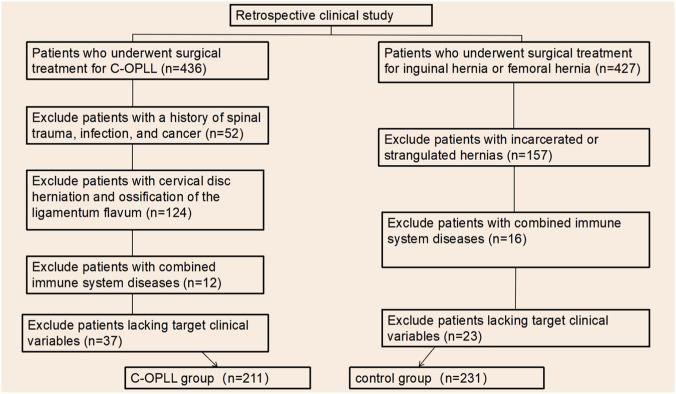
Patient inclusion and exclusion flowchart.

Patients who underwent surgical treatment for C-OPLL at our institution between January 2021 and December 2024 were consecutively enrolled in the C-OPLL group. Considering that inflammatory and immune dysregulation have been implicated in the pathogenesis of degenerative musculoskeletal disorders, and that traumatic conditions may induce acute systemic inflammatory responses, patients who underwent elective hernia repair for reducible inguinal or femoral hernia during the same period were selected as the control group. This control population was chosen to minimize potential confounding effects related to trauma- or degeneration-associated systemic inflammation. The following patients were excluded: Patients with a history of trauma, autoimmune diseases (e.g., ankylosing spondylitis or rheumatoid arthritis), infection, or malignancy; Patients diagnosed with cervical disc herniation or ossification of the ligamentum flavum; Patients presenting with incarcerated or strangulated hernia; Patients with incomplete or missing clinically relevant data. The diagnosis of C-OPLL was made based on typical radiographic findings on cervical CT and MRI (Figure 1). Following the inclusion and exclusion screening, the total number of patients enrolled in the final analysis was 442. Among them, 211 were patients with C-OPLL, whereas 231 were control patients (Figure 2).

### 2.3 Demographic and clinical data collection

The variables collected from each patient enrolled in the study included sex, age, BMI, smoking and drinking status, comorbidities such as osteoporosis, hypertension, diabetes, cardiovascular disease, liver disease, history of stroke, and pre-operative peripheral blood cell counts, including neutrophils, platelets, monocytes and lymphocytes.

### 2.4 Inflammatory indices calculation

Inflammation-related indices were calculated as follows:

NLR = neutrophil count/lymphocyte count.

PLR = platelet count/lymphocyte count.

MLR = monocyte count/lymphocyte count.

SII = platelet count × neutrophil count/lymphocyte count.

Peripheral blood samples were obtained preoperatively as part of routine clinical evaluation.

### 2.5 Statistical analysis

Normality of continuous variables was assessed using the Kolmogorov–Smirnov test. Between-group comparisons were performed using the independent-samples t-test or Mann–Whitney U test for continuous variables, and the chi-square test for categorical variables. Univariate and multivariate logistic regression analyses with backward stepwise elimination were conducted to identify independent risk factors for C-OPLL. Variables with P < 0.05 in univariate analysis were entered into the multivariate model. In the multivariate logistic regression analysis, the SII was modeled as a continuous variable scaled per 100-unit increase, and BMI was modeled per 1 kg/m^2^ increase. Multicollinearity among independent variables was evaluated using the variance inflation factor (VIF), with values above 5 indicating significant collinearity. Results are expressed as odds ratios (ORs) with 95% confidence intervals (CIs). ROC curve analysis was performed to analyze the discriminative ability of inflammatory biomarkers and the optimal cut-off value was determined using the Youden index.

To evaluate the consistency of SII and C-OPLL association across demographic and clinical characteristics, stratified subgroup analyses were performed with the inclusion of their interaction terms to evaluate effect modification. Pearson correlation coefficients were calculated to evaluate correlations among inflammatory indices, visualized as a heatmap. To examine the synergistic effect of metabolic burden and systemic inflammation, patients were further stratified by BMI (Chinese criteria: normal weight, overweight, obese) (Fan et al., 2025) and SII cutoff values. Logistic regression was employed to compute ORs for each combined subgroup against the reference of normal weight + low SII. In the model, a multiplicative interaction term (BMI category × SII) was added to evaluate interaction. All statistical analyses were carried out using SPSS (version 20.0) and R software (version 3.5.0). A two-tailed P < 0.05 was considered statistically significant.

## 3 Results

### 3.1 Baseline characteristics

In the final analysis, a total of 442 patients (211 in the C-OPLL group and 231 in the control group) were included. As shown in Table 1, the C-OPLL group was considerably older than the control group (60.30 ± 3.25 vs. 55.19 ± 7.71, p < 0.05), and patients in the C-OPLL group had a higher BMI (26.13 ± 3.14 vs. 23.87 ± 3.31, p < 0.01). Apart from these findings, no significant differences were observed in other demographic or clinical characteristics between the two groups.

**TABLE 1:** Demographics for patients.

Variable	C-OPLL group (N = 211)	Control group (N = 231)	p Value
Age (years)	60.30 ± 3.25	55.19 ± 7.71	0.018*
Sex, n (%)			0.062
Male	130 (61.61%)	122 (52.81%)	
Female	81 (38.39%)	109 (47.19%)	
BMI (kg/m2)	26.13 ± 3.14	23.87 ± 3.31	0.001*
Smoking, n (%)			0.140
Yes	76 (36.02%)	68 (29.44%)	
No	135 (63.98%)	163 (70.56%)	
Alcohol, n (%)			0.975
Yes	88 (41.71%)	96 (41.56%)	
No	123 (58.29%)	135 (58.44%)	
Comorbidities on admission, n (%)			0.828
Osteoporosis	21 (9.95%)	16 (6.93%)	
Diabetes	33 (15.64%)	28 (12.12%)	
Hypertension	54 (25.59%)	61 (26.41%)	
Cardiovascular disease	21 (9.95%)	25 (10.82%)	
Liver disease	12 (5.69%)	13 (5.63%)	
Stroke	9 (4.27%)	7 (3.03%)	

Statistically significant data are indicated by an asterisk (*) and bold formatting.

### 3.2 Inflammatory biomarkers

Regarding inflammatory biomarkers, Table 2 illustrates that preoperative NLR and SII were significantly elevated in the C-OPLL group compared with controls (p < 0.05), whereas PLR and MLR did not differ significantly between groups (p > 0.05). Besides, no significant differences were observed between the two groups regarding preoperative laboratory values, including serum albumin and total cholesterol, platelet count, lymphocyte count, monocyte count, and neutrophil count.

**TABLE 2:** Preoperative laboratory data and immune inflammatory index.

Variable	C-OPLL group (N = 211)	Control group (N = 231)	p Value
Serum albumin (g/dL)	37.45 ± 4.46	37.07 ± 4.73	0.329
Total cholesterol (mmol/L)	3.23 ± 0.17	3.26 ± 0.18	0.157
Platelet count	221.62 ± 67.66	218.11 ± 67.63	0.912
Lymphocyte count	1.94 ± 0.67	1.86 ± 0.69	0.130
Monocyte count	0.39 ± 0.16	0.38 ± 0.17	0.106
Neutrophil count	4.15 ± 2.27	4.01 ± 3.16	0.242
NLR	2.58 ± 2.72	2.05 ± 1.08	0.039*
PLR	127.43 ± 61.94	137.89 ± 80.88	0.136
MLR	0.22 ± 0.10	0.24 ± 0.18	0.637
SII	638.15 ± 481.89	487.59 ± 300.34	0.001*

Statistically significant data are indicated by an asterisk (*) and bold formatting.

### 3.3 Univariate and multivariate logistic regression analyses

Univariate logistic regression analysis identified age, BMI, NLR, and SII as variables significantly associated with the presence of C-OPLL (p < 0.05). Variables with p < 0.05 in the univariate analysis were subsequently entered into the multivariate logistic regression model using backward stepwise elimination. Multivariate analysis demonstrated that both BMI and SII were independent risk factors for C-OPLL. For every 1-unit increase in BMI, the risk increased by 41.2% (OR = 1.412, 95%CI: 1.251–1.594, p < 0.01); for every 100-unit increase in SII, the risk increased by 12.1% (OR = 1.121, 95%CI: 1.101–1.210, p < 0.01). After adjustment for confounding variables, NLR was no longer statistically significant (p > 0.05). Table 3 presents the full results of the regression.

**TABLE 3:** Univariate and multivariate logistic regression analysis.

Variable	Univariate			Multivariate		
	OR	95% CI	p Value	OR	95% CI	p Value
Age (years)	1.032	1.006–1.059	0.016*	1.000	0.970–1.030	0.977
BMI (kg/m2)	1.242	1.164–1.326	0.001*	1.412	1.251–1.594	0.001*
NLR	1.192	1.030–1.326	0.019*	1.044	0.925–1.178	0.483
SII	1.001	1.000–1.001	0.007*	1.121	1.101–1.210	0.006*

Statistically significant data are indicated by an asterisk (*) and bold formatting. Abbreviations: OR, odds ratio; CI, confidence interval. Odds Ratio (per 100-unit increase in SII/per 1 kg/m^2^ increase in BMI).

### 3.4 Predictive performance of SII and BMI

**FIGURE 3:**
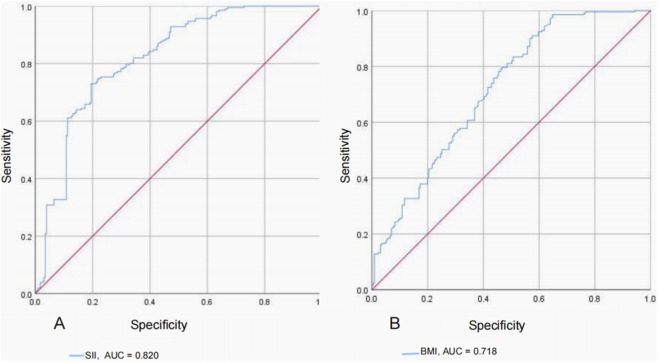
Receiver Operating Characteristic (ROC) curve for systemic immune-inflammation index (SII) **(A)** and body mass index (BMI) **(B)** in C-OPLL. The X-axis represents specificity, and the Y-axis represents sensitivity.

ROC curve analysis was performed to evaluate the discriminative ability of SII for predicting C-OPLL. Of the various indices evaluated, SII was found to possess superior predictive power to BMI, with an area under the curve (AUC) of 0.82 (95% CI: 0.78–0.86, p < 0.001) indicating good discriminatory power (Figure 3). The optimal cutoff value of SII is 464.2 by the maximum Youden index analysis which has a sensitivity and a specificity of 73.0% and 80.5%.

### 3.5 Subgroup analysis

**FIGURE 4:**
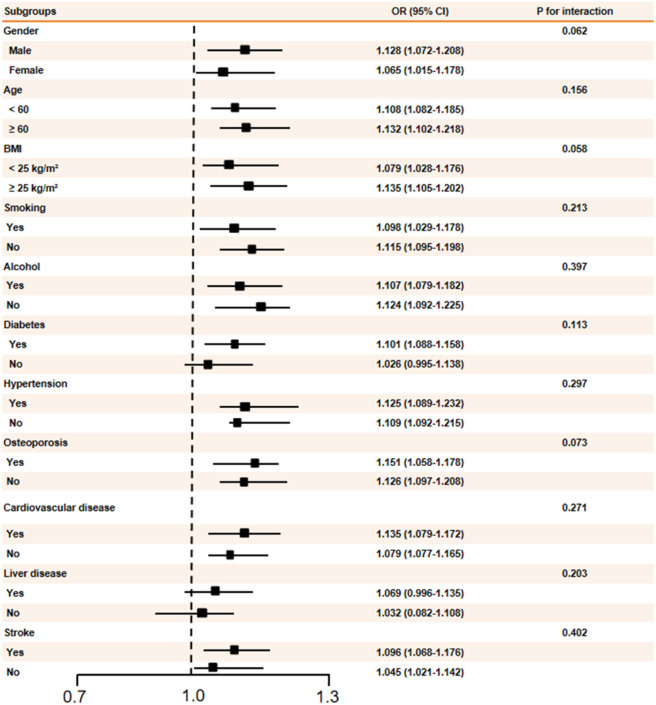
Subgroups analysis and interaction analysis for SII in C-OPLL.

To evaluate whether the predictive value of SII for C-OPLL varied by demographic or clinical characteristics, we performed subgroup analyses stratified by sex, age, BMI, smoking, alcohol consumption, and common comorbidities. The forest plot (Figure 4) showed a persistent positive correlation between SII and C-OPLL across every subgroup with ORs ranging from 1.026 to 1.151. Tests for interaction showed no evidence of statistical significance for effect modification by any of the subgroup variables (all P > 0.05) but the interaction with gender (P = 0.062) and BMI (P = 0.058) were close. These findings indicate that the association between SII and C-OPLL is robust and not substantially influenced by these factors.

### 3.6 Correlation among inflammatory indices

**FIGURE 5:**
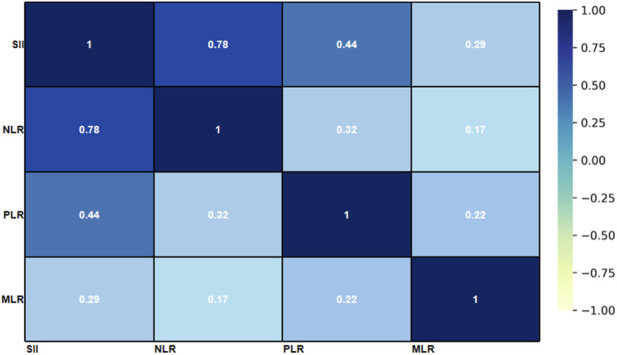
Correlation heatmap of inflammatory indices (SII, NLR, PLR, and MLR). The color intensity reflects the magnitude of correlation (warmer colors indicate positive correlation; cooler colors indicate negative correlation).

The correlations among SII, NLR, PLR, and MLR are presented in the heatmap (Figure 5). A strong positive correlation was observed between SII and NLR (r = 0.78, P < 0.001) and a moderate correlation between SII and PLR (r = 0.44, P < 0.001). A weaker correlation was observed between SII and MLR (r = 0.29, P < 0.01). As per these results, we confirm that SII incorporates information from a mixture of inflammatory cell lineages; Hence, SII may serve as a composite marker of systemic inflammation.

### 3.7 Synergistic effect of BMI and SII on C-OPLL risk

**FIGURE 6:**
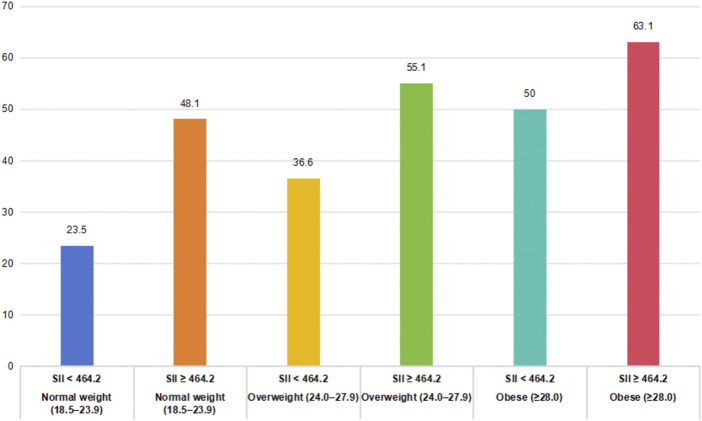
Prevalence of C-OPLL stratified by BMI categories and SII levels.

Table 4 and Figure 6 show the combined effect of BMI and SII on C-OPLL risk, stratified by both variables. Of the 442 patients, prevalence of C-OPLL increased progressively within the subgroups of BMI and SII (P for trend <0.001). Relative to the reference group (normal weight + low SII, prevalence: 23.5%), the prevalence was 48.1% in the normal-weight + high-SII group, 36.6% in the overweight + low-SII group, 55.1% in the overweight + high-SII group, 50.0% in the obese + low-SII group, and 63.1% in the obese + high-SII group.

**TABLE 4:** Synergistic effect of BMI (Chinese criteria) and SII on C-OPLL risk.

BMI Category	SII Group	Total (N)	C-OPLL (N)	Prevalence (%)	OR (95% CI)	P value
Normal weight (18.5–23.9)	SII <464.2	68	16	23.5	1.00 (reference)	
Normal weight (18.5–23.9)	SII ≥464.2	52	25	48.1	3.02 (1.42–6.41)	0.004
Overweight (24.0–27.9)	SII <464.2	82	30	36.6	1.88 (0.95–3.71)	0.069
Overweight (24.0–27.9)	SII ≥464.2	78	43	55.1	4.00 (2.01–7.96)	<0.001
Obese (≥28.0)	SII <464.2	40	20	50	3.26 (1.47–7.22)	0.004
Obese (≥28.0)	SII ≥464.2	122	77	63.1	6.23 (3.3–11.26)	<0.001*

*P for interaction (BMI category × SII) = 0.038*.

Abbreviations: BMI, body mass index; SII, systemic immune-inflammation index; OR, odds ratio; CI, confidence interval.

Notably, patients with both obesity and elevated SII presented increased risk with an odds ratio approximately 1.9-fold higher than that of the obese + low-SII group and 1.6-fold higher than that of the overweight + high-SII group. Formal testing showed a significant BMI category × SII interaction (P for interaction = 0.038), indicating that the effect of SII on C-OPLL risk was significantly modified by BMI status. The findings suggest a synergistic effect of metabolic burden and systemic inflammation in the development of C-OPLL, with maximum risk in individuals having obesity and systemic inflammation together.

## 4 Discussion

In this retrospective study of 442 patients, we observed SII and BMI as independent risk factors for C-OPLL. Compared with other inflammatory indices (NLR, PLR, and MLR), SII remained an independent predictor after multivariable adjustment and showed better discriminative performance (AUC 0.82), suggesting its potential added value as a composite systemic marker. This finding is consistent with prior reports in spinal ossification disorders, where SII was associated with ligament ossification (Zhao et al., 2022)^.^ These findings suggest that both metabolic burden and systemic inflammatory activation may be related to the development of aberrant ligament ossification.

The present study demonstrates that BMI is an independent risk factor of C-OPLL, which is in close agreement with conclusions of recent large-sample clinical studies. Kobashi et al. (2004) reported that BMI was significantly higher in the OPLL group than in the non-OPLL group, regardless of gender. Similarly, Hirai et al. (2016) further observed a positive correlation between BMI and ossification index in OPLL patients (P < 0.05), suggesting that higher BMI is not only associated with OPLL risk but may also influence disease severity. A cohort study including 622 asymptomatic patients with OPLL also showed that the obese group had a significantly higher rate of OPLL than the control group, showing an independent association of BMI with OPLL (Endo et al., 2022). Notably, recent research found that the OPLL prevalence rate in obese populations characterized by increased visceral fat was 2–2.5 times higher than in the control group, and visceral fat accumulation may be closely related to ossification progression (Fukada et al., 2023; Sato et al., 2025), indicating that fat distribution patterns may play a more significant role in OPLL progression.

The epidemiological correlation between BMI or obesity and OPLL has been relatively well-studied; however, the biological mechanisms have yet to be fully elucidated. According to current research, there might be several pathways involved. First, adipose tissue is an active secretory and endocrine organ, whose major secreted peptide leptin is increased significantly in OPLL patients (Feng et al., 2018). Experimental studies have demonstrated that leptin promotes the proliferation and osteogenic differentiation of osteoblasts and chondrocytes through ERK1/2, MAPK, STAT3, and JNK signaling pathways (Feng et al., 2018; Chen et al., 2018). The low-grade inflammation linked to obesity is not to be neglected (Kawaguchi et al., 2017; Ren et al., 2013). The cytokines released by adipose tissue are C-reactive protein, tumour necrosis factor-α, and interleukin-6. It is possible that the local microenvironment is modified by these mediators. Increased body mass may enhance the mechanical stresses on the cervical spine posterior longitudinal ligament due to the increased load acting on it. This may result in osteogenic differentiation of the ligament cells, which is manifested by the upregulation of osteogenesis-related genes and increased alkaline phosphatase activity (Zhu et al., 2023). In addition, the hyperglycemia and hyperinsulinemia that are metabolic comorbidities of obesity may induce the osteoblastic differentiation of spinal ligament cells by promoting production of reactive oxygen species (ROS), activating protein kinase C (PKC) isoforms, and upregulating the BMP2/Runx2 and PI3K/Akt signaling pathways (Bai et al., 2005; Chen et al., 2006; Li et al., 2008). These findings may provide molecular clues that help us understand the link between obesity and OPLL, although further mechanistic studies are required to determine direct causation.

Immune microenvironment in inflammation can regulate the osteoblast proliferation and differentiation process of OPLL (Osta et al., 2014). As a composite inflammatory marker that integrates neutrophil, platelet, and lymphocyte counts, the SII provides a broader reflection of the body’s inflammatory immune status compared with single inflammatory parameters. Moreover, the SII has shown high predictive value for adverse clinical events in various diseases. The inflammatory mechanisms involved in heterotopic ossification have become more of a focus lately. Earlier research on inflammatory factors in OPLL has demonstrated that the local tissues and serum of OPLL patients show significantly elevated levels of these inflammatory factors. This level is closely related to the size of the lesions and rate of progression (Li et al., 2022). Nonetheless, the data available on composite markers of systemic inflammation including SII is scanty. This research is the first to show that SII is an independent risk factor for C-OPLL, which provides a novel inflammatory immunological pathogenesis (He et al., 2022). It should be noted that inflammatory cells do play a direct promoting role in exogenous ossification (Herath et al., 2018; Herath et al., 2021). The authors Herath et al. determined that neutrophils can significantly enhance osteoblasts osteogenic activity, and they observed that neutrophils can promote new bone formation in a rabbit bone defect model (Herath et al., 2018; Herath et al., 2021).

Recent epidemiological studies have provided increasing evidence for the involvement of inflammatory mediators in OPLL pathogenesis. Yayama et al. reported an increment in serum levels of IL-6, IL-1α and fibroblast growth factor in OPLL patients (Yayama et al., 2022). Furthermore, Saito et al. showed increased expression of IL-6 in the spinal ligament tissue, indicating that chronic inflammation is involved in the pathogenesis of heterotopic ossification (Saito et al., 2023). Wu et al. (2025) provided direct evidence of systemic inflammation in OPLL by showing significantly increased expression of pro-inflammatory cytokines (IL-1β, TNF-α, MCP-1) in peripheral blood monocytes, alongside markedly reduced levels of the anti-inflammatory cytokine IL-10. Notably, Takahata et al. in a nation-wide multicenter case-control study found significantly high serum TNF-α concentration in OPLL patients comparing to the controls (Takahata et al., 2025). This observation was still significant even after careful matching with controls for age, sex, and BMI. The authors, therefore, suggest that systemic inflammatory activation may be involved in the pathogenesis independent of metabolic derangement. According to another review, OPLL should be considered a systemic pathological state closely associated with the endocrine-metabolic disorder instead of a simple local degenerative change (Wang et al., 2025).

The systemic inflammatory state represented by SII could act in concert with other pathways to influence OPLL progression. As the main immune cells in the body, neutrophils release reactive oxygen species and pro-inflammatory cytokines that aggravate the inflammatory cascade. When neutrophils are hyperactivated, there is an increased formation of neutrophil extracellular traps (NETs) (Nunez et al., 2023). This, in turn, activates the bone morphogenetic protein (BMP) and transforming growth factor-β (TGF-β) signaling pathways, thereby encouraging the osteogenic differentiation of mesenchymal stem cells (Nunez et al., 2023; Shu et al., 2024). According to prior study (Fujii et al., 2025), platelets may also directly contribute to bone regulation through the production of platelet-derived growth factor (PDGF) and TGF-β. Ranganathan et al. showed that lymphocyte-deficient mice had dramatically reduced heterotopic ossification suggesting an important role for lymphocytic infiltration in its pathogenesis (Fujii et al., 2025; Ranganathan et al., 2016). According to Zhao et al. (2022), high SII levels were independent predictors of thoracic ossification of the ligamentum flavum (OR = 12.16) and were positively correlated with ossification scores which are indicative of systemic inflammatory activation as a pathogenic mechanism of spinal ligament ossification. Chronic low-grade inflammation associated with obesity may activate signalling pathways like ERK1/2 and MAPK which induces osteogenic differentiation (Kawaguchi et al., 2017; Ren et al., 2013). This suggests that BMI and SII can exert cumulative effects through a common signalling pathway which is an inflammatory one. Taken together, the increase of SII may reflect the existence of a systemic pro-osteogenic inflammatory microenvironment that promotes ligamentous heterotopic ossification, providing a theoretical basis for future anti-inflammatory interventions.

In clinical practice, BMI and SII represent inexpensive, readily accessible markers that could enhance risk stratification for C-OPLL. The availability of these biomarkers helps in the early detection of high-risk individuals, which can allow closer monitoring and timely clinical assessment before the occurrence of symptoms.

There are several limitations to consider. The single-center retrospective design is subject to concern of selection bias limiting generalizability. In addition, single-timepoint measurement of inflammatory markers precludes the evaluation of longitudinal dynamics and causality. The use of hernia patients as controls rather than healthy community controls may further affect external validity, as baseline systemic inflammatory and metabolic statuses may differ between groups despite multivariable adjustment. Furthermore, as an observational study, our data cannot establish causality between SII/BMI and C-OPLL. Finally, although we discussed plausible biological mechanisms, the current study did not directly test how systemic inflammation may promote ossification of the posterior longitudinal ligament. It is therefore important that prospective multicenter studies and experimental investigations are done to confirm these observations and provide biological mechanisms.

## 5 Conclusion

This study demonstrates that the SII and BMI are independent predictive factors for C-OPLL, revealing the important roles of inflammatory and metabolic factors in the pathogenesis of this disease. Since these two indicators are easily obtainable and can be used for risk stratification and early identification, they provide important reference value for subsequent in-depth research on their mechanisms of action and the development of intervention studies for early prevention of C-OPLL.

## Data Availability

The raw data supporting the conclusions of this article will be made available by the authors, without undue reservation.
